# A multi-platform comparison and machine learning associative analysis study on the information dissemination effect of short videos for cerebral hemorrhage: A cross-sectional study based on TikTok and Bilibili

**DOI:** 10.1097/MD.0000000000050408

**Published:** 2026-08-21

**Authors:** Can Luo, Xiong Deng, JieYao Xia, Song Tian

**Affiliations:** a The First Affiliated Hospital of Shaoyang University, Shaoyang, Hunan, China.

**Keywords:** cerebral hemorrhage, content quality, health communication, machine learning, short videos

## Abstract

Cerebral hemorrhage is a critical public health issue marked by high incidence, disability, and mortality. In China, short-video platforms have become the major channel for the public to acquire relevant health knowledge. However, the online cerebral hemorrhage-related health information varies greatly in quality, with complex dissemination mechanisms and a lack of systematic academic evaluation, which greatly weakens the authenticity and practicality of popular science content for the public. This study aimed to compare the content ecosystems of cerebral hemorrhage-related short videos on TikTok and Bilibili, explore the core factors associated with video dissemination effects, and evaluate the classification performance and feature correlation of machine learning models in identifying high-quality health popularization videos. A total of 200 cerebral hemorrhage-related short videos were collected from the 2 platforms, with their metadata, interaction indicators, and content quality scores recorded. Statistical tests and regression analyses were adopted to compare platform differences and variable correlations. Nine machine learning models were built for high-quality video identification. Area under the curve, calibration curves, and ridgeline plots were used to evaluate model performance and visualize the distribution characteristics of core research indicators. The 2 platforms presented obvious differences in content ecological characteristics. Bilibili had longer-duration, institution-produced, and higher-quality videos, while TikTok featured short videos, individual creation, and stronger user interactivity. Light Gradient Boosting Machine showed the optimal analytical performance, and video duration was the core stable feature affecting video quality. Videos created by medical professionals had significant advantages in information credibility, actionability, and understandability. This study identified a notable “quality-dissemination paradox,” revealing the essential conflict between platform algorithm logic and public health information quality. Nonmedical creators need to enhance professional capabilities, and platforms should optimize recommendation algorithms to boost the efficient and accurate dissemination of high-quality cerebral hemorrhage health information.

## 1. Introduction

Stroke ranks as the second leading cause of human mortality globally. Epidemiological data demonstrate that from 1990 to 2019, stroke prevalence increased by approximately 850%, while mortality increased by approximately 430%. Among stroke subtypes, cerebral hemorrhage accounts for 27.9% of all stroke cases.^[1]^ Cerebral hemorrhage is defined as nontraumatic bleeding caused by the rupture of intracranial blood vessels in the brain parenchyma. This condition has a high acute mortality rate, with only approximately one-fifth of patients achieving functional independence after onset, imposing substantial economic and caregiving burdens on affected families and society.^[2]^ Notably, compared with high-income countries, the peak incidence of cerebral hemorrhage in China occurs in the mid-1960s, approximately 1 decade earlier than the mid-1970s peak observed in high-income countries.^[3]^ Lifestyle modifications and metabolic disorder management are modifiable risk factors with significant preventive value for cerebral hemorrhage. Accordingly, lifestyle intervention and targeted public health education serve as core strategies for the primary prevention of this disease.^[4,5]^ In China, cerebral hemorrhage imposes a severe epidemiological burden, affecting millions of individuals each year,^[6,7]^ which highlights an urgent need for standardized public health education to improve population literacy regarding cerebral hemorrhage. Evidence-based health information dissemination can effectively enhance public prevention awareness, improve early symptom recognition, standardize prehospital emergency procedures, clarify clinical intervention schemes, and guide personalized rehabilitation management. Short-video platforms, represented by TikTok and Bilibili, have replaced traditional media and search engines as the dominant health information sources for young populations, benefiting from their intuitive visual presentation, ultrafast dissemination efficiency, and massive user coverage.^[8]^ However, while these platforms accumulate abundant cerebral hemorrhage-related short-video resources, the quality of such health content varies drastically, accompanied by widespread misinformation and misleading health guidance risks.^[9–11]^ Moreover, unique algorithmic recommendation mechanisms and user interaction modes of short-video platforms reshape traditional health information dissemination pathways, which may hinder the effective propagation of high-quality medical and health content.^[12–14]^ To date, few studies have systematically compared the content quality of cerebral hemorrhage-related short videos across mainstream short-video platforms. Additionally, machine learning approaches have been insufficiently applied to explore the multidimensional associative relationships between video characteristics and information dissemination effects. This cross-sectional study was designed to fill these research gaps, with 2 core research dimensions and refined specific objectives. The first dimension focuses on systematic content quality evaluation, while the second dimension targets the exploration of platform-specific dissemination associations, with no causal inference performed.

The specific research objectives are as follows: to systematically characterize and compare the differences in content characteristics, author profiles, and standardized content quality of cerebral hemorrhage-related short videos on TikTok and Bilibili; to explore the key associative factors of multidimensional user interaction and dissemination indicators across the 2 platforms; to construct multiple machine learning models to analyze the associative correlations between video features and high-quality health content, and compare the analytical performance of different models; to deconstruct the multidimensional attributes of video content quality based on 3 authoritative evaluation systems (Global Quality Scale [GQS], modified DISCERN [mDISCERN], Patient Education Materials Assessment Tool [PEMAT]) and clarify the intrinsic association between content quality and short-video dissemination effects. Notably, this study adopts a strict cross-sectional design. All analyses in this study are limited to associative correlation exploration, and no causal inferences are drawn. The findings of this study aim to provide empirical evidence for understanding the quality-dissemination paradox of cerebral hemorrhage health communication on short-video platforms and offer practical references for health content creators, platform managers, and public health institutions to optimize health information dissemination.

## 2. Method

### 2.1. Data collection and sampling

**Figure 1.**
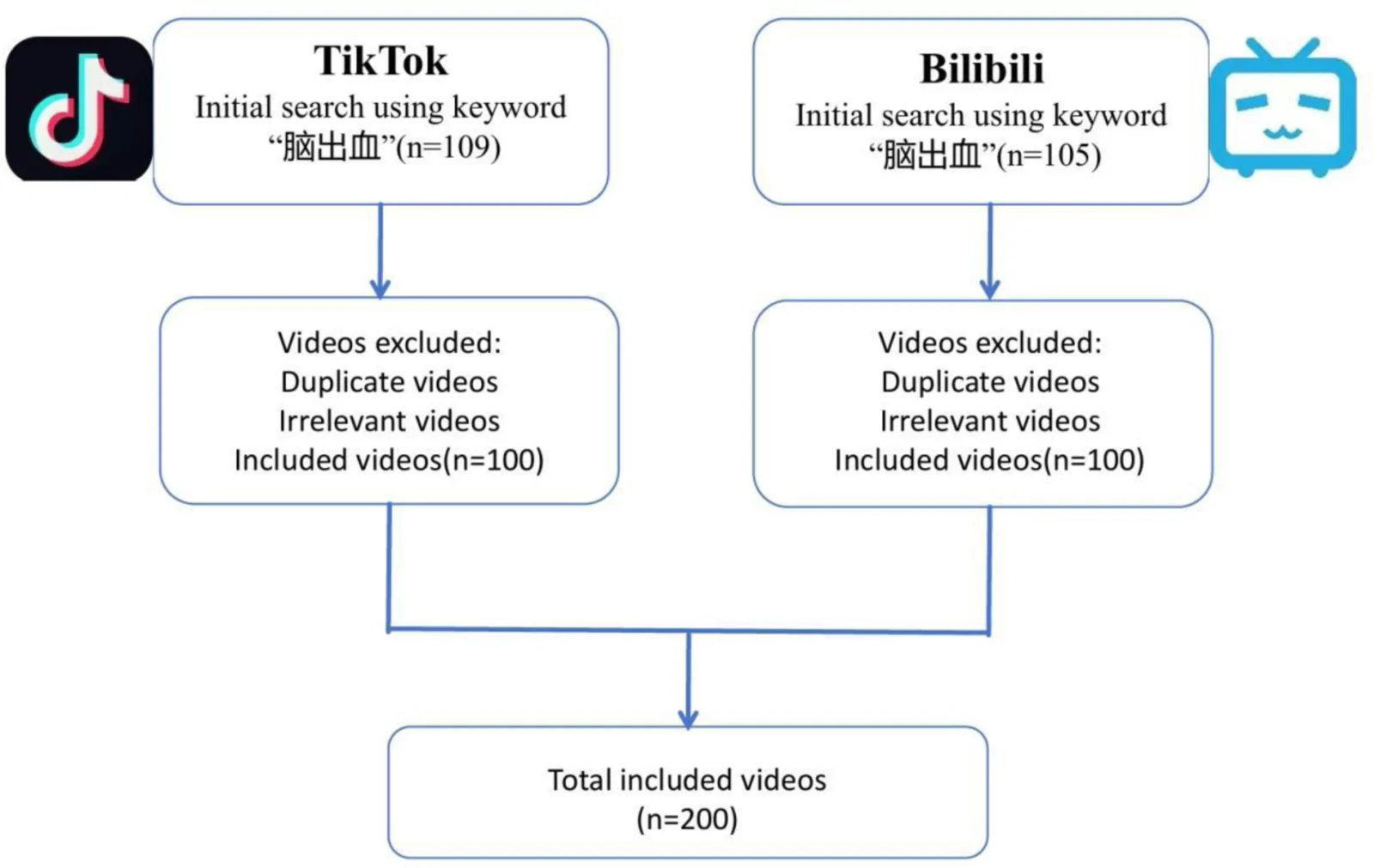
Search strategy for videos on intracerebral hemorrhage. n = number of videos.

This investigation employed a cross-sectional study design. To effectively reduce algorithmic selection bias caused by personalized platform recommendation mechanisms, data collection on both TikTok and Bilibili was conducted using newly registered neutral accounts with no prior browsing or viewing history, ensuring that video retrieval was not affected by individual algorithmic preferences. All video resources and corresponding metadata were obtained based on public and open-access channels of the 2 platforms. The unified search term “cerebral hemorrhage” was used for video retrieval, and all included videos were required to be released at least 1 month before data collection to guarantee sufficient stabilization of interaction data. A standardized and reproducible sampling screening procedure was strictly implemented throughout the study. Original short videos focusing on cerebral hemorrhage science popularization, symptom identification, disease prevention, emergency treatment, and rehabilitation guidance were included, while duplicate content, purely commercial promotional videos, irrelevant thematic content, low-quality fragmented clips, and videos with incomplete interaction data were excluded. According to previous relevant short-video health communication studies and effect size estimation, a minimum of 80 videos per platform was sufficient to meet basic statistical requirements. To further improve data stability, correlation reliability, and machine learning model training performance, the sample size was appropriately expanded. Initial searches yielded 109 videos from TikTok and 105 from Bilibili; after rigorous exclusion, 100 valid cerebral hemorrhage-related short videos were finally selected from each platform, resulting in a total sample of 200 videos (Fig. 1).

### 2.2. Variable definition and measurement

Metadata: video duration, release date, and author follower count.

Interaction data: number of likes, comments, shares, and collections.

Content types: classified into 5 categories: popular science education, advertising/commercial promotion, case sharing/patient narratives, news media reports, and others.

Diagnosis and treatment dimension: categorized into 12 disease-related themes based on health information content, including causes/risk factors, diagnostic methods, disease prevention, symptom recognition, treatment plans, and others.

Author identities: classified into 6 categories: medical institutions, medical professionals, individual creators (without medical backgrounds), patients/family members sharing personal experiences, commercial health accounts, and media/news organizations.

Content quality: manual coding and scoring were conducted utilizing 3 standardized, validated assessment tools: GQS: A 5-point scale assessing overall perceived quality of health information^[15]^; simplified DISCERN scale (mDISCERN): a 5-point scale evaluating information credibility and evidence citation practices^[16]^; PEMAT: comprising 2 subscales (PEMAT-Understandability [PEMAT-U] and PEMAT-Actionability [PEMAT-A]) expressed as percentage scores, measuring content comprehensibility and practical applicability.^[17]^

Dissemination effect: the 4 interaction indicators, including likes, comments, shares, and collections, were integrated to construct a multidimensional comprehensive communication effect index. For binary classification modeling, the 75th percentile of platform-specific comprehensive interaction data was set as the cutoff value. Videos with comprehensive interaction volume above the threshold were defined as high-dissemination popular videos,^[1]^ and those below the threshold were defined as ordinary videos (0). Each engagement metric was equally weighted in the composite index after logarithmic transformation.

Video quality: operationalized as PEMAT composite score; for machine learning analysis, videos with both PEMAT-U and PEMAT-A scores exceeding 75% were classified as “high-quality videos,”^[1]^ while those not meeting both thresholds were classified as “non-high-quality videos” (0).

All video content coding and quality scoring were completed independently by 2 researchers. In case of discrepancies in their scores, a third researcher would conduct an additional evaluation to ensure the objectivity and reproducibility of the subjective scoring results. We calculated intraclass correlation coefficient and Cohen Kappa to evaluate inter-rater consistency. Values above 0.8 for both coefficients indicated good agreement. Given the real-time updating feature of online short videos, a priori sample size calculation and power analysis were not conducted. All videos were screened following standardized procedures. The final sample size is adequate to support content assessment, reliability analysis, and subsequent machine learning associative analyses, and the samples are representative of the target content on both platforms.

### 2.3. Analysis process

Descriptive and differential analysis: frequency and percentage distributions were calculated for categorical variables. The chi-square test was employed to compare distribution differences between platforms for categorical variables (content type, author identity, diagnosis-treatment dimension). Independent samples *t*-test was utilized to compare mean differences between platforms for continuous variables (interaction metrics, quality scores, metadata).

Correlation and regression analysis: binary logistic regression and linear regression models were performed to explore the core associative correlates of video dissemination performance. The outcome variable was defined as video popularity status based on the comprehensive multidimensional dissemination index. All non-normally distributed continuous variables were logarithmically transformed before regression analysis. Categorical video content types were ordinally encoded according to the degree of medical relevance for subsequent regression modeling. Consistent with the cross-sectional study design, all regression analyses were used for correlation exploration rather than causal inference or analytical modeling.

Correlation analysis: Spearman rank correlation coefficient was calculated to explore overall association patterns among all key continuous variables (metadata, interaction metrics, quality scores), with results visualized through heatmap representation.

Machine learning modeling: data with large differences in orders of magnitude were subjected to standardization processing. Least absolute shrinkage and selection operator regression (within the logistic regression framework, family = binomial) was used to screen 9 candidate features, including likes, favorites, comments, shares, duration, author followers, and all dimensions of mDISCERN, GQS, and PEMAT. The optimal penalty parameter *λ* was selected through 10-fold cross-validation. Final incorporated features were determined based on the minimum mean squared error criterion (*λ*.min) and the 1-standard-error rule (*λ*.1se). Using the filtered features, 9 machine learning algorithms were compared to evaluate model performance: Extreme Gradient Boosting, logistic regression, random forest, Light Gradient Boosting Machine (LightGBM), Adaptive Boosting, decision tree, Gradient Boosting Decision Tree, Support Vector Machine, and Gaussian Naive Bayes. Random data splitting (70% for training and 30% for testing) was repeated 5 times, and the area under the curve (AUC) with 95% confidence intervals (CI) was calculated on the test set. Model overfitting was identified by comparing AUC values between the training and test sets. Calibration curves were generated to assess probability calibration accuracy, and feature importance rankings were calculated to identify key factors associated with video quality.

Quality score distribution visualization: Ridgeline plots were constructed for each quality assessment tool (GQS, mDISCERN, PEMAT-A, PEMAT-U) to visually depict distribution patterns of quality scores across author identity categories, content types, and diagnosis-treatment dimensions.

All statistical analyses were conducted using Python 3.9 with scikit-learn and statsmodels libraries. Data visualization was accomplished using Matplotlib and Seaborn libraries.

### 2.4. Ethics approval

This study did not involve the use of clinical data, human specimens, or laboratory animals. All information was sourced from publicly available videos on TikTok and BiliBili, with no inclusion of personal privacy data. Furthermore, as there was no interaction with platform users, ethical review was not required.

## 3. Results

### 3.1. Sample description and platform differences

A total of 200 videos underwent analysis. Independent samples *t*-test analysis revealed a notable “platform paradox”: TikTok videos demonstrated significantly shorter duration, yet all interaction metrics (likes, comments, shares, collections) and author follower counts substantially exceeded those on Bilibili (*P* < .01). Conversely, Bilibili videos demonstrated significantly superior performance across all content quality measures (GQS, PEMAT-U, PEMAT-A, mDISCERN) compared with TikTok (*P* < .01). Table 1

**Table 1:** TikTok vs Bilibili platform comparison.

Variable	TikTok	Bilibili	*P*
Release date	12.21 ± 6.55	12.58 ± 6.81	.631
Duration (sec)	189.75 ± 34.91	520.04 ± 76.76	.000*
Shares	641.12 ± 1568.66	287.47 ± 359.98	.032*
Favorites	15,188.67 ± 6397.78	7239.40 ± 1995.51	.000*
GQS	3.24 ± 0.87	4.16 ± 0.76	.000*
PEMAT-Understandability	0.61 ± 0.14	0.86 ± 0.10	.000*
PEMAT-Actionability	0.63 ± 0.18	0.83 ± 0.14	.000*
mDISCERN	3.03 ± 1.04	4.11 ± 0.95	.000*
Likes	16,259.79 ± 6441.20	6280.75 ± 1771.45	.000*
Comments	13,050.42 ± 5096.07	5405.69 ± 1452.12	.000*
Author followers	293,518.55 ± 227015.99	158,395.62 ± 67849.45	.000*

GQS = Global Quality Scale, mDISCERN = modified DISCERN, PEMAT = Patient Education Materials Assessment Tool.

* *P* < .05.

Chi-square test analysis demonstrated highly significant differences in content type distribution (*χ*^2^ = 27.67, *P* < .001) and author identity distribution (*χ*^2^ = 36.56, *P* < .001) between platforms. Bilibili content demonstrated concentrated focus on “popular science education” (91.0%), with primary contributions from “medical institutions” (39.0%). TikTok content exhibited greater diversity, including “popular science education” (62.0%), “case sharing” (11.0%), “advertising/commercial promotion” (9.0%), and other categories, with primary authorship from “medical professionals” (52.0%). No significant platform differences emerged in diagnosis-treatment dimension distribution (*P* > .05), with most prevalent themes being disease prevention (20.0%), symptom recognition (15.5%), and rehabilitation nursing (12.0%). Table 2

**Table 2:** TikTok vs Bilibili platform comparison.

Variable		TikTok (n [%])	Bilibili (n [%])	*P*
Content type	Advertisement/commercial promotion	9 (9.0)	0 (0.0)	.000*
	Case sharing/patient narrative	11 (11.0)	5 (5.0)	
	News/media report	9 (9.0)	4 (4.0)	
	Others	9 (9.0)	0 (0.0)	
	Science popularization education	62 (62.0)	91 (91.0)	
Disease dimension	Cause and risk factors	10 (10.0)	10 (10.0)	.967
	Diagnostic differentiation	2 (2.0)	2 (2.0)	
	Diagnostic methods	10 (10.0)	6 (6.0)	
	Disease prevention	19 (19.0)	21 (21.0)	
	Emergency treatment	9 (9.0)	11 (11.0)	
	Medication guidance	2 (2.0)	2 (2.0)	
	Others	1 (1.0)	2 (2.0)	
	Prognosis and recurrence prevention	9 (9.0)	11 (11.0)	
	Rehabilitation and care	10 (10.0)	14 (14.0)	
	Symptom recognition	18 (18.0)	13 (13.0)	
	Treatment options	10 (10.0)	8 (8.0)	
Author type	Commercial health accounts	9 (9.0)	0 (0.0)	.000*
	Individual creator (non medical_background)	9 (9.0)	6 (6.0)	
	Media/news organizations	8 (8.0)	0 (0.0)	
	Medical institution	11 (11.0)	39 (39.0)	
	Medical professional	52 (52.0)	51 (51.0)	
	Patient/family share	11 (11.0)	4 (4.0)	

n = number of videos.

* *P* < .05.

The inter-rater reliability between the 2 coders was satisfactory, with all variables yielding intraclass correlation coefficient or Cohen Kappa values > 0.85 (Table S1, Supplemental Digital Content 1).

### 3.2. Video release time

**Figure 2.**
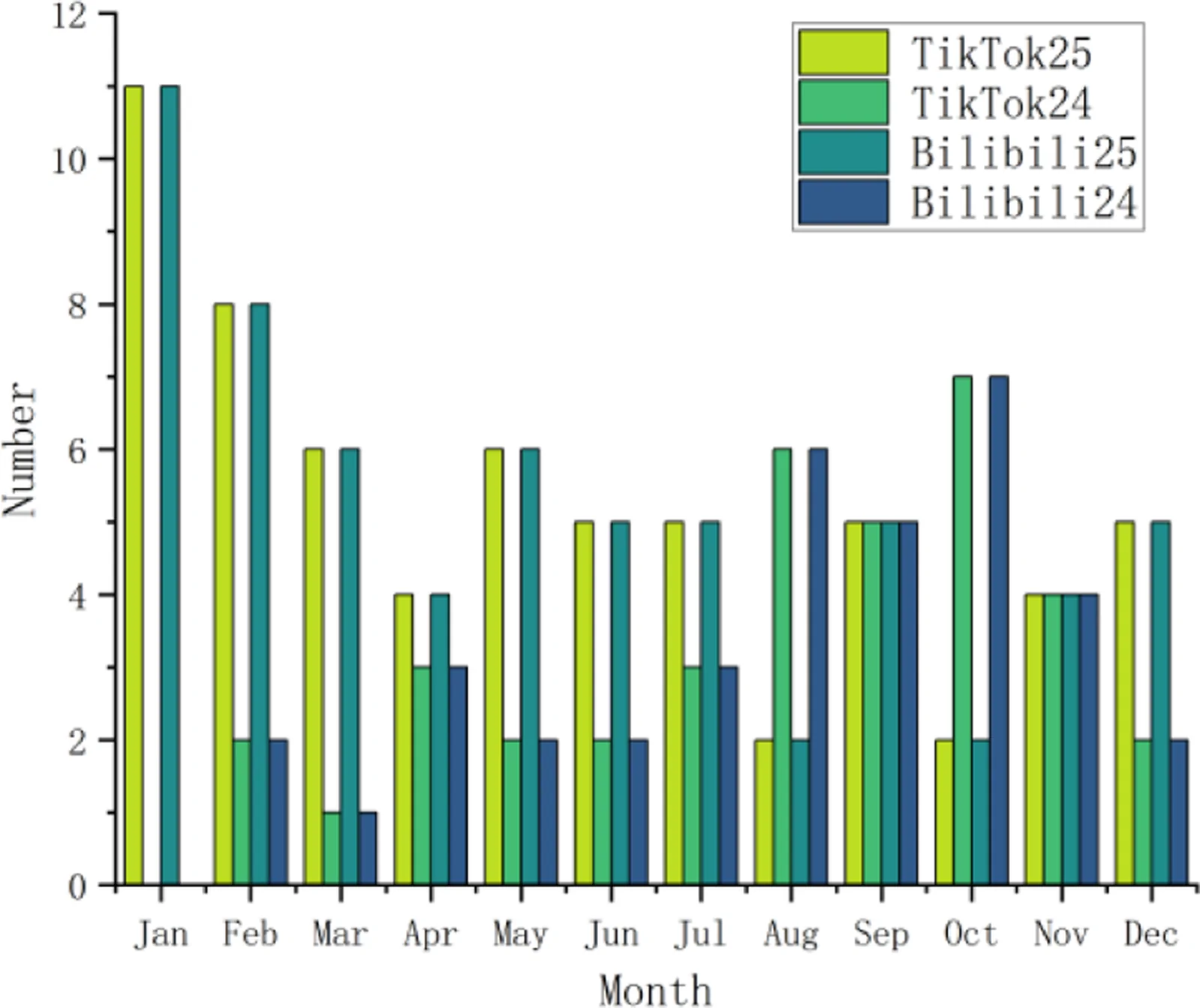
Bar chart of video release time

Most videos were posted in January. The overall temporal distribution of video releases was relatively flat with minor fluctuations. The number of videos posted on TikTok and Bilibili in 2025 was higher than that in 2024. Figure 2

### 3.3. Key factors influencing dissemination effect

**Figure 3.**
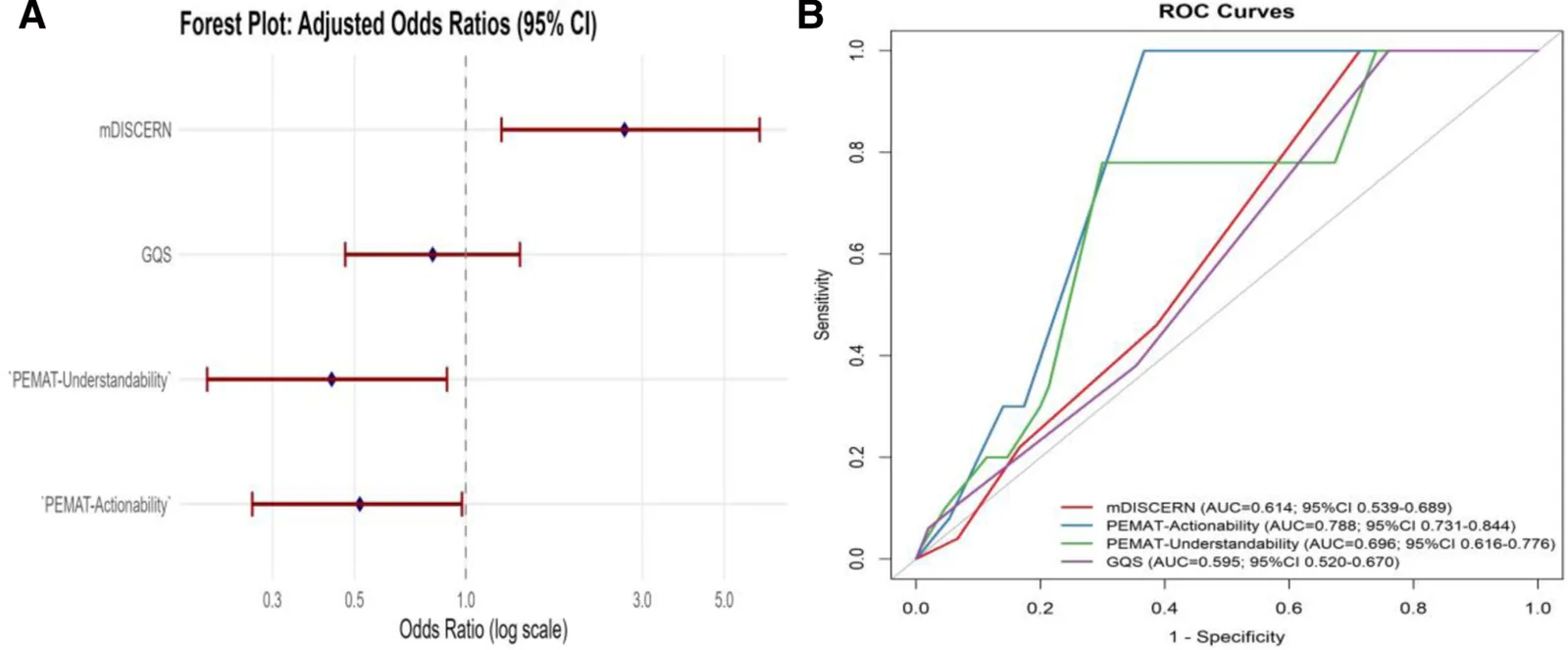
(A) Relative risks of variables for videos becoming viral (forest plot); (B) area under the characteristic curve of variables for viral short videos. AUC = area under the curve, CI = confidence interval, GQS = Global Quality Scale, mDISCERN = modified DISCERN, PEMAT = Patient Education Materials Assessment Tool, ROC = receiver operating characterisitc.

Binary logistic regression analysis (Table 3, Fig. 3) showed that mDISCERN was significantly positively associated with a high comprehensive communication effect (odds ratio [OR] = 2.68, 95% CI: 1.25–6.23, *P* = .015). Conversely, PEMAT-A (OR = 0.52, 95% CI: 0.26–0.98, *P* = .046) and PEMAT-U (OR = 0.43, 95% CI: 0.20–0.89, *P* = .027) were significantly negatively associated with comprehensive communication effect. GQS showed no significant association (*P* > .05).

**Table 3:** Key results summary of binary logistic regression analysis for high-interaction short videos.

Variable	Regression coefficient	OR	95% CI	*P*
mDISCERN	0.98	2.68	1.25–6.23	.015*
PEMAT-Actionability	−0.65	0.52	0.26–0.98	.046*
PEMAT-Understandability	−0.83	0.43	0.20–0.89	.027*
GQS	−0.20	0.81	0.47–1.40	.455

CI = confidence interval, GQS = Global Quality Scale, mDISCERN = modified DISCERN, OR = odds ratio, PEMAT = Patient Education Materials Assessment Tool.

* *P* < .05.

Receiver operating characteristic analysis demonstrated that the AUC values were 0.614 for mDISCERN, 0.788 for PEMAT-A, 0.696 for PEMAT-U, and 0.595 for GQS, all above 0.5 but with modest discriminative performance.

**Figure 4.**
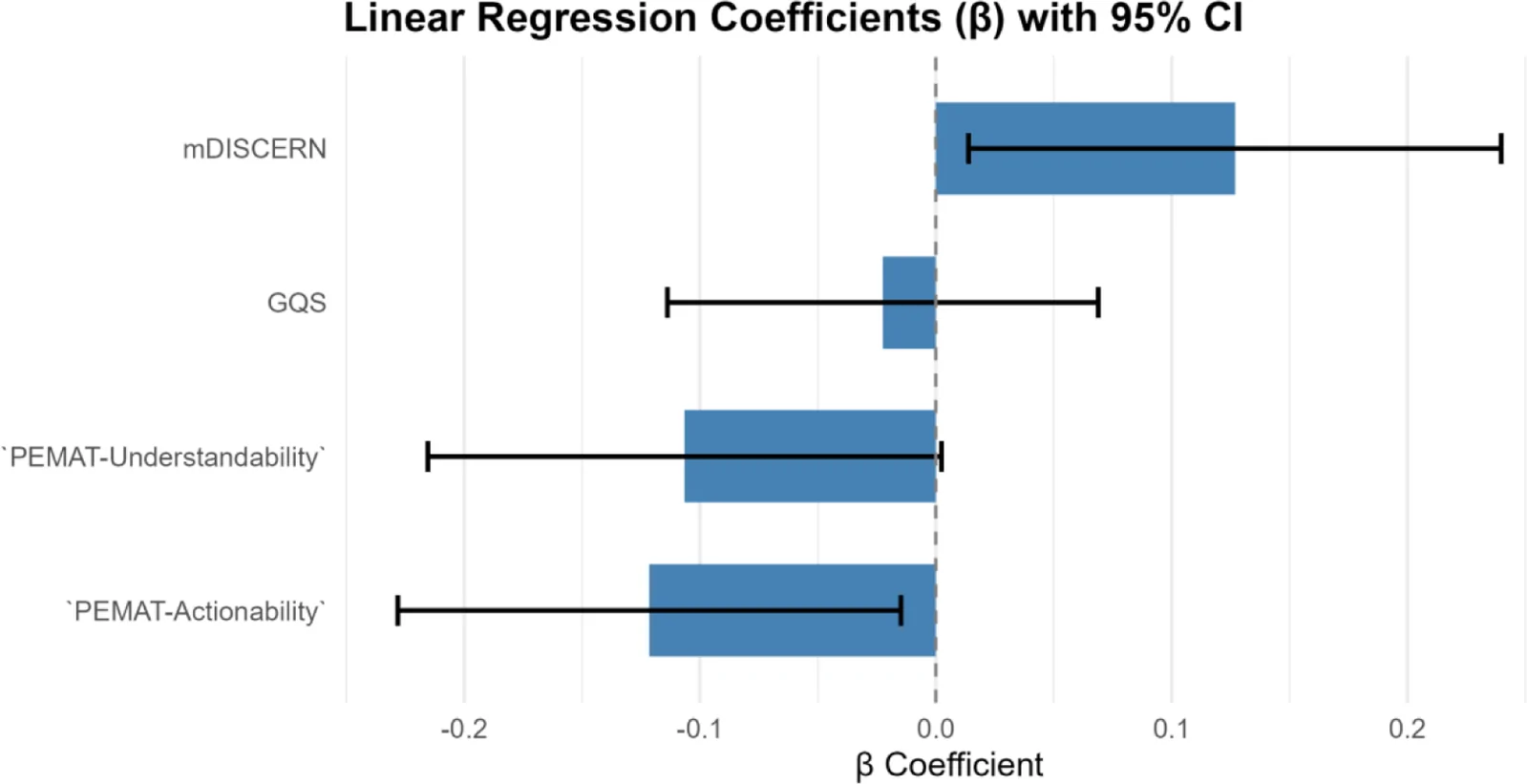
Comparison of the relative influence of each variable on popular videos. CI = confidence interval, GQS = Global Quality Scale, mDISCERN = modified DISCERN, PEMAT = Patient Education Materials Assessment Tool.

Linear regression analysis (Table 4, Fig. 4) yielded consistent results. mDISCERN was positively associated with comprehensive communication effect (*β* = 0.13, *P* = .03), while PEMAT-A was negatively associated (*β* = −0.12, *P* = .03). PEMAT-U showed a marginally significant negative association (*β* = −0.11, *P* = .05). GQS showed no significant association (*P* > .05).

**Table 4:** Linear regression results for video interaction performance.

Variable	*β*	SE	Standardized *β*	*t*	*P*
mDISCERN	0.13	0.057	0.13	2.21	.03*
PEMAT-Actionability	−0.12	0.054	−0.12	−2.25	.03*
PEMAT-Understandability	−0.11	0.055	−0.11	−1.93	.05
GQS	−0.02	0.046	−0.02	−0.49	.62

GQS = Global Quality Scale, mDISCERN = modified DISCERN, PEMAT = Patient Education Materials Assessment Tool, SE = standard error.

* *P* < .05.

### 3.4. Correlation analysis

**Figure 5.**
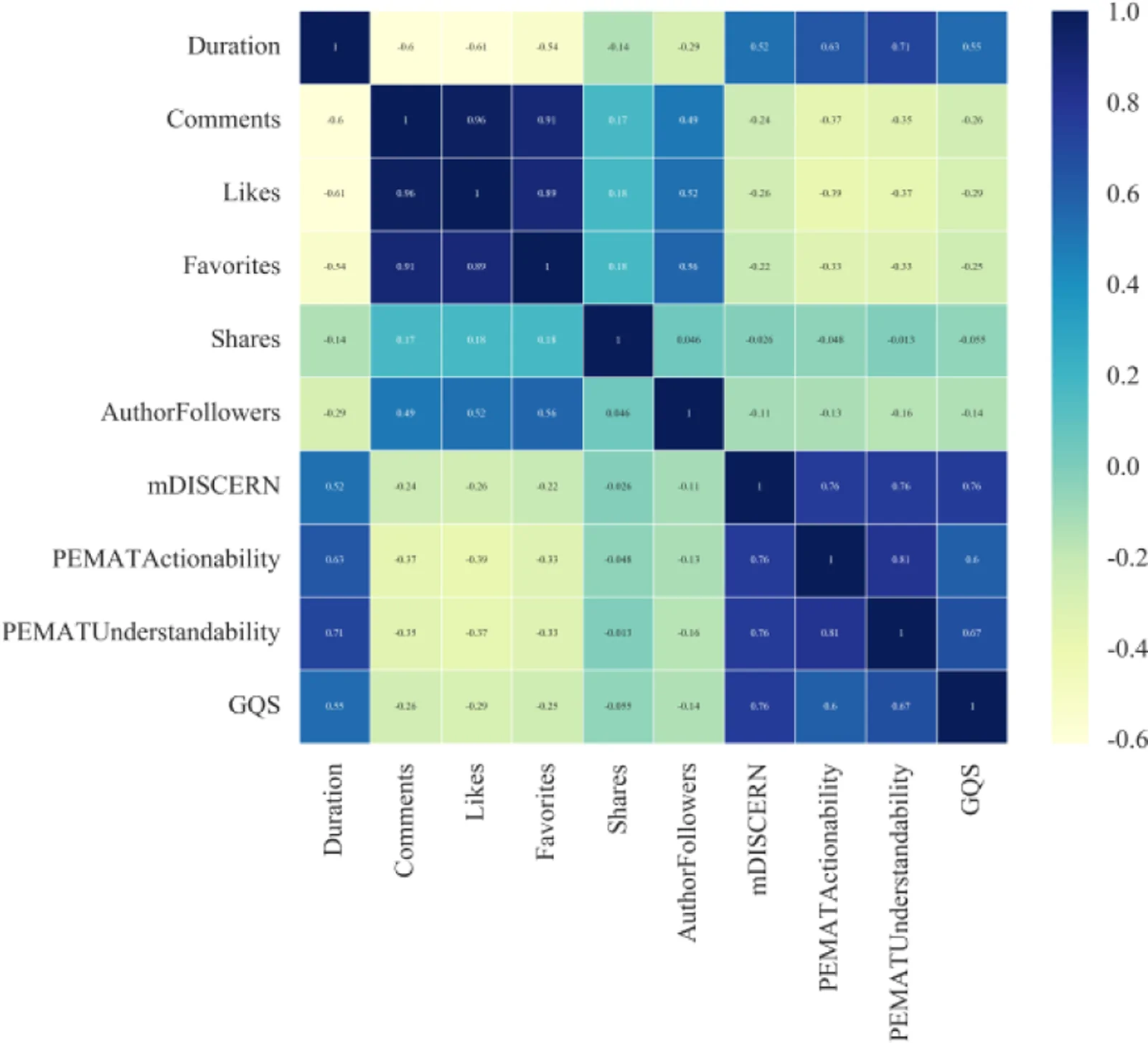
Spearman rank correlation heatmap of key variables (metadata, interaction metrics, quality scores). GQS = Global Quality Scale, mDISCERN = modified DISCERN, PEMAT = Patient Education Materials Assessment Tool.

To comprehensively elucidate interrelationships among variables affecting dissemination effect, Spearman rank correlation analysis was performed, generating a heatmap encompassing metadata, interaction indicators, and quality scores (Fig. 5). The correlation heatmap revealed positive associations between all interaction metrics (likes, comments, shares, collections) and author follower count, indicating that creators’ online influence is closely linked to user engagement. Content quality scores (GQS, PEMAT-A, PEMAT-U, mDISCERN) were positively correlated with video duration. Notably, content quality scores showed negative correlations with interaction indicators, suggesting that higher-quality videos do not necessarily gain proportionally greater user engagement.

### 3.5. Comparison of model analytical performance

Least absolute shrinkage and selection operator regression was used to screen factors associated with high-quality videos. Likes, shares, and video duration were retained, whereas all other variables were penalized to 0. Among the 9 machine learning models, tree-based and ensemble models exhibited far better overall performance than linear and kernel-based models.

**Figure 6.**
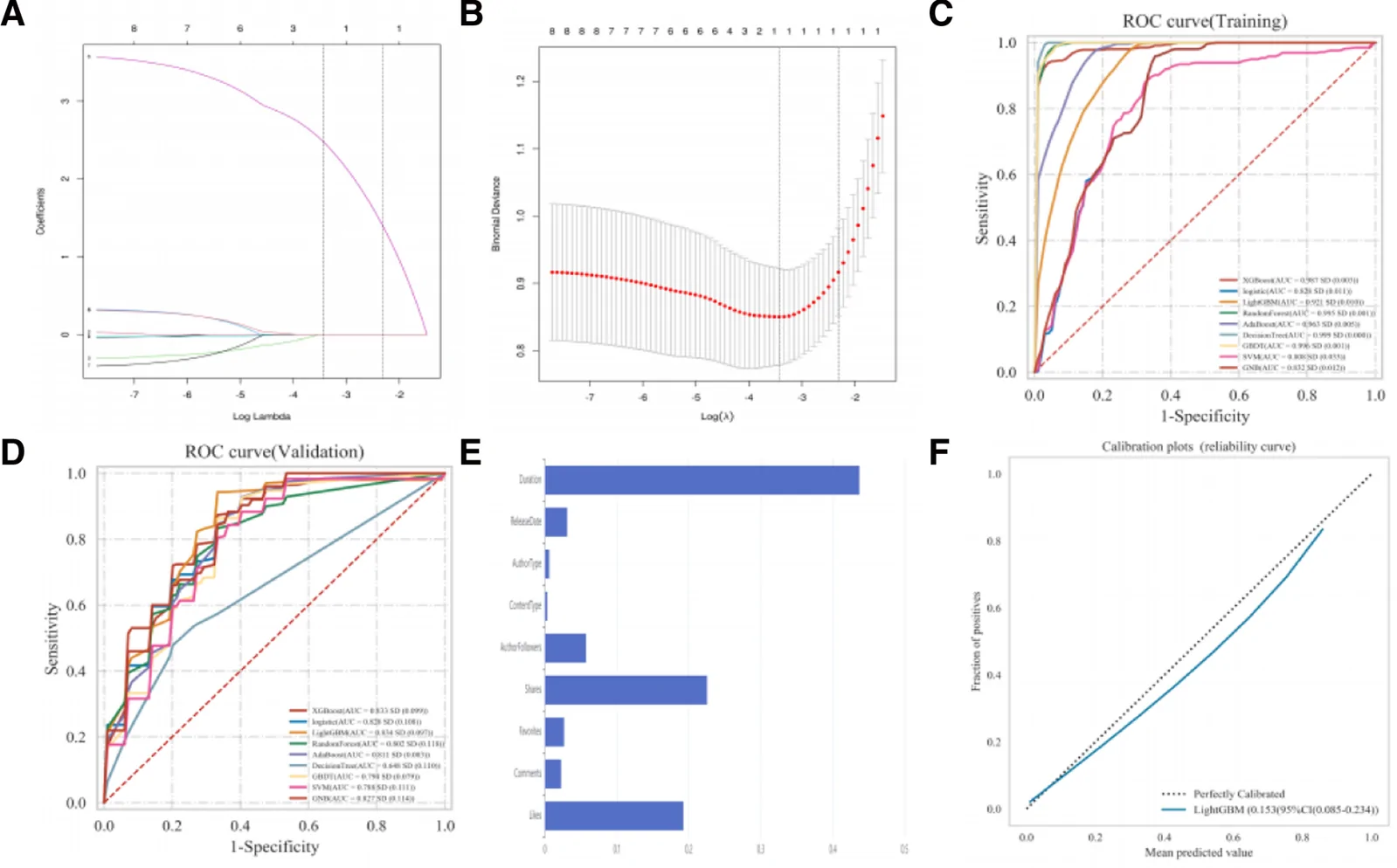
(A) Coefficient profile plot of lasso regression; (B) cross-validation curve plot of LASSO regression; (C) ROC curve for machine learning training; (D) ROC curve for machine learning validation; (E) bar chart of feature importance; (F) calibration curve of LightGBM. LASSO = least absolute shrinkage and selection operator, LightGBM = Light Gradient Boosting Machine, ROC = receiver operating characterisitc.

Noticeable differences in generalization capacity were observed across models in 10-fold cross-validation. Tree-based and ensemble models generally achieved high performance on the training set; yet, most of them suffered varying degrees of performance decline on the validation set. The Decision Tree Classifier showed an obvious performance drop, suggesting severe overfitting. By contrast, linear and probabilistic models maintained relatively stable but moderate performance across training and validation sets. Among all candidates, the LightGBM Classifier delivered the most stable results and favorable generalizability. Calibration analysis further verified that LightGBM produced more reliable probability estimates across validation folds. Based on multiple evaluation metrics including AUC, F1-score, accuracy, precision, and recall (Table S2, Supplemental Digital Content 2), LightGBM achieved the optimal overall performance among all 9 machine learning models. It obtained the highest AUC value of 0.834, indicating the strongest discriminative ability between high-dissemination and ordinary short videos. Meanwhile, LightGBM maintained balanced precision and recall, as well as satisfactory generalization capacity. Extreme Gradient Boosting showed comparable performance and served as an alternative model. By contrast, the single decision tree suffered from severe overfitting with a low AUC of 0.648, and Gaussian Naive Bayes presented unbalanced performance with excessively high recall. According to feature importance analysis based on tree-based models, video duration was the leading factor correlated with PEMAT scores (Fig. 6).

### 3.6. Multidimensional distribution visualization of content quality

**Figure 7.**
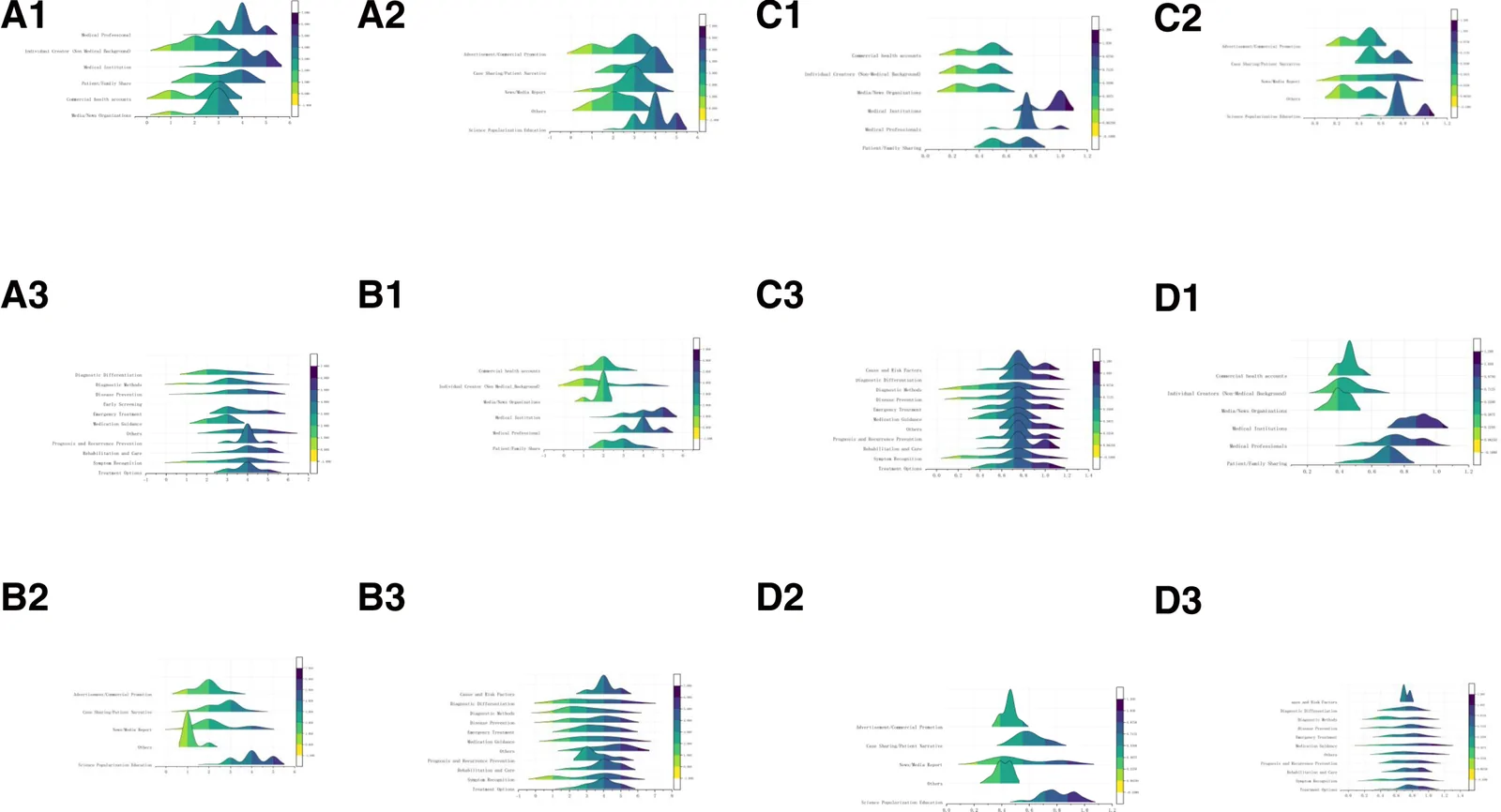
(A) Ridge plot of health short-video quality distribution based on GQS scores: author identity, content type, disease dimension; (B) ridge plot of health short-video quality distribution based on mDISCERN scores: author identity, content type, disease dimension; (C) ridge plot of health short-video quality distribution based on PEMAT-Actionability scores: author identity, content type, disease dimension; (D) ridge plot of health short video quality distribution based on PEMAT-Understandability scores: author identity, content type, disease dimension. GQS = Global Quality Scale, mDISCERN = modified DISCERN, PEMAT = Patient Education Materials Assessment Tool.

Ridgeline plot visualization was performed to intuitively illustrate the multidimensional distribution characteristics of content quality scores across different author identities, video content types, and medical thematic dimensions. In terms of the GQS and mDISCERN quality evaluation systems, videos produced by medical professionals and official medical institutions, well as videos categorized as popular science education content, exhibited concentrated and right-skewed score distributions, representing the highest overall content quality levels among all groups. In contrast, videos focusing on disease diagnosis and treatment showed relatively uniform and stable score distributions with limited numerical fluctuations. Regarding the PEMAT-A and PEMAT-U dimensions, which respectively assessed content actionability and understandability, videos created by professional medical creators and popular science educational content consistently achieved superior evaluation performance. Similarly, the quality scores of videos related to disease diagnosis and treatment were evenly distributed without obvious differentiation across the PEMAT assessment dimensions. All visualized distribution patterns clearly reflected the structural heterogeneity of video content quality in different subgroups (Fig. 7).

## 4. Discussion

### 4.1. Comparative analysis of TikTok and Bilibili ecosystems

Our findings confirm that TikTok videos exhibit significantly shorter durations while demonstrating substantially higher performance across all interaction metrics (likes, comments, shares, collections) and author follower counts relative to Bilibili. Conversely, Bilibili videos demonstrated significantly superior content quality scores (GQS, PEMAT-U, PEMAT-A, mDISCERN) compared with TikTok. This phenomenon may be associated with fundamental differences in platform architectures and algorithmic design. TikTok’s competitive advantage in health communication may be associated with its “short and concise” content delivery approach and recommendation algorithm incorporating collaborative filtering and content-based analysis.^[18,19]^ Within the cerebral hemorrhage information domain, this architecture enables precise targeting of popular science short videos to users with relevant search histories or demonstrated information interests. Evidence demonstrates that TikTok effectively delivers concise, visually compelling information addressing adolescent mental health or acute disease prevention.^[20]^ For disseminating cerebral hemorrhage early recognition information (e.g., sudden headache, limb weakness), TikTok’s abbreviated video format proves exceptionally suited for rapidly conveying essential information points, exemplified by “Stroke Recognition” protocols.^[21]^ Additionally, TikTok’s global expansion and the substantial user base of its Chinese iteration (Douyin) have facilitated the accumulation of billions of cerebral hemorrhage-related video views,^[22]^ enabling medical experts through this “public traffic pool” model to rapidly establish personal professional brands and transform complex pathophysiological concepts into accessible visual representations.^[23]^ Conversely, Bilibili’s information ecosystem emphasizes “longer-form content + in-depth analysis.”^[24]^ Bilibili users typically demonstrate stronger intrinsic motivation for learning, accounting for the platform’s popularity for educational live streams (exemplified by “Study With Me” formats) and evidence-based scientific content.^[25]^ Within cerebral hemorrhage information dissemination, Bilibili videos frequently incorporate detailed anatomical illustrations, surgical procedure demonstrations (including decompressive craniectomy techniques), and extended rehabilitation training modules spanning tens of minutes.^[26,27]^ Bilibili’s distinctive “danmu” (bullet comment) culture introduces real-time interactive dimensions to health communication, enabling users to express illness-related concerns or contextual medical terminology commentary in a synchronous fashion. This bidirectional communication simultaneously alleviates patient family anxiety and establishes informal electronic support networks.^[28]^ The “quality–dissemination paradox” identified in this study can be theoretically explained from 3 interrelated perspectives: health information accessibility theory, mediatization logic, and algorithmic distribution theory. From the perspective of health communication, high-quality cerebral hemorrhage popular science content strictly adheres to professional medical norms and forms a standardized, rigorous, and scientific content system that meets professional quality control requirements. However, such professionally oriented content conforms to supply-side medical standards but contradicts the public’s cognitive patterns of health information reception. Audiences generally follow the cognitive least-effort principle and prefer fragmented, visually intuitive, and easy-to-understand short-video content. Consequently, high-quality professional health information often carries higher cognitive thresholds, which may make it difficult to match public daily media consumption habits. From the perspective of media communication logic, short-video platforms operate on a traffic-oriented principle, and algorithmic recommendation mechanisms prioritize user interactive metrics such as likes, comments, and shares rather than content professionalism and academic quality. Platform algorithms place low weight on medical accuracy and normative value, potentially resulting in a misalignment between media dissemination logic and public health popularization goals. High-quality professional medical content with substantial public health value may not obtain rapid early-stage user engagement, whereas simplified, low-professionalization popular science content may better align with algorithmic preferences and public entertainment demands, thereby potentially gaining greater traffic exposure. The inherent conflict between professional health quality governance and platform algorithmic traffic logic may contribute to the structural paradox whereby higher content quality corresponds to lower dissemination performance, reflecting the core imbalance between information supply and public demand in short-video health communication. Platform recommendation algorithms prioritize early-stage user interaction signals (likes) when determining video entry into “trending” traffic tiers, while intrinsic content quality receives insufficient algorithmic weighting. This pattern reflects a dominant “traffic-first” logic, wherein high-quality content failing to generate substantial early engagement encounters barriers to achieving broad dissemination.^[29,30]^

### 4.2. Comparative analysis of health information quality regarding cerebral hemorrhage

The academic literature routinely employs standardized assessment instruments, including GQS, PEMAT, and mDISCERN, to evaluate health education video quality.^[31,32]^ These instruments provide objective quality quantification across dimensions encompassing information reliability, comprehensibility, practical utility, and production quality. Multiple cross-disciplinary investigations have documented that cerebral hemorrhage-related videos on TikTok demonstrate exceptionally rapid dissemination rates, though with notable variability in quality stability. On one hand, short videos created by certified medical professionals achieve relatively high accuracy in health advice (AUC scores).^[33,34]^ For instance, acute cerebral hemorrhage management videos by practicing physicians on TikTok frequently articulate concise critical directives such as “avoid self-medication” and “activate emergency medical services promptly.”^[26]^ Conversely, TikTok’s inherent format constraints frequently compromise the completeness of medical background information. Videos posted by nonprofessional creators may deliberately oversimplify disease etiology or exaggerate dietary preventive effects on cerebral hemorrhage to optimize engagement metrics, constituting acknowledged vectors for “health misinformation” propagation.^[35]^ Parallel investigations examining analogous fields, including thyroid nodules and spinal cord injuries, have similarly documented that average quality scores of TikTok videos correlate positively with creator professionalism, yet the low entry barriers for nonprofessional creators generate substantial overall quality variance.^[36,37]^ Bilibili offers markedly more in-depth cerebral hemorrhage videos, with mean video durations substantially exceeding TikTok equivalents. This temporal allocation permits comprehensive treatment of cerebral hemorrhage classification (hypertensive vs amyloidosis subtypes), imaging manifestations (computed tomography hyperdensity patterns), and comparative analysis of surgical approaches with respective advantages and limitations.^[38]^ Paradoxically, investigations within certain medical domains (brain tumors, myocardial infarction, etc) reveal that overall Bilibili video reliability scores occasionally fall below TikTok equivalents.^[22,39]^ Comparative research indicates both TikTok and Bilibili have evolved into essential platforms for hospital-based public health education. Predominant “influential physician” (big V) accounts on TikTok typically employ oral presentation formats, producing content during personal time. Representative videos addressing myocardial infarction and uremia cases frequently serve as engagement vehicles, subsequently directing audience attention toward cerebral hemorrhage prevention knowledge.^[39,40]^ These productions characteristically demonstrate template-based, high-frequency dissemination patterns. In contrast, professional creators on Bilibili pursue specialized, team-based production models. Official cerebral hemorrhage videos from prominent tertiary healthcare institutions on Bilibili typically feature high-production-value post-processing and documentary-style presentation approaches. Although this “refinement” strategy generates lower output frequency, it cultivates more enduring audience trust and institutional brand recognition.

### 4.3. Practical implications

For professional content creators and medical institutions, strengthening “knowledge translation” competencies represents an urgent priority. While preserving scientific rigor, these creators should master short-video narrative grammar and emotional expression techniques to bridge the dichotomy between professionalism and public appeal. For platform and algorithm developers, more sophisticated recommendation architectures warrant development, integrating authoritative source signals, multidimensional quality metrics, and positive engagement indicators to prevent algorithmic marginalization of high-quality public health information. Currently, algorithms may preferentially promote content generating elevated user engagement and completion rates, frequently favoring cerebral hemorrhage videos featuring dramatic titles and impactful visuals (surgical scenes with blood imagery, extreme survival narratives) while relegating methodical, evidence-based prevention guidance to obscurity.^[18,41]^ Although Bilibili algorithms similarly operate under engagement-driven logic, platform search functionality assumes greater prominence in user information-seeking behavior. Users typically conduct goal-directed searches (e.g., “How to manage acute cerebral hemorrhage”), and this search-centric information acquisition pattern partially mitigates the drawbacks of passive fragmented content consumption.^[38,39]^ False health information on short-video platforms exhibits multi-pathway dissemination characteristics. Although Bilibili similarly contains misinformation, its active danmu “error correction mechanism” generates natural quality filtration. When obvious medical factual errors appear in videos, experienced users or medical students frequently mark these inaccuracies through real-time bullet comments, thereby reducing misleading content impact.

### 4.4. Limitations and future research directions

This investigation presents several methodological limitations. First, the cross-sectional design captures video data at a single temporal point, limiting the ability to characterize dynamic changes in information flow regarding cerebral hemorrhage. Second, existing research predominantly addresses the “supply side” of short videos (content production), with a notable lack of “demand-side” research (user behavioral modifications following video consumption). Third, as a heterogeneous medical phenomenon, the differential platform representation of cerebral hemorrhage subdomains (subarachnoid hemorrhage, cerebellar hemorrhage, etc) warrants comprehensive exploration. Fourth, although the GQS, mDISCERN, and PEMAT scales are validated and widely adopted in health communication studies, these generic tools may not fully capture short-video-specific attributes such as audiovisual narrative characteristics, algorithm-driven visibility, and engagement-oriented content design. Furthermore, given that online short videos are dynamically updated, we did not perform a priori sample size calculation and power analysis prior to data collection, which also constitutes a limitation of the present work. Future investigations should prioritize multimodal learning approaches for in-depth analysis of large-scale short-video datasets. Simultaneously, cross-cultural comparative research should be expanded to characterize similarities and distinctions between TikTok’s international and Chinese (Douyin) versions regarding popular science preferences for cerebral hemorrhage content. From a governance perspective, developing real-time detection algorithms for identifying erroneous health information in short videos represents a critical priority for maintaining health information ecosystem integrity.

### 4.5. Conclusion

This multi-method investigation reveals the “quality-dissemination paradox” and inherent tension between “professionalization-popularization” objectives characterizing short-video health communication. Research findings demonstrate that optimizing the short-video health information ecosystem cannot rely exclusively on user interaction metrics. Rather, it necessitates collaborative efforts among platforms, content creators, and researchers to construct integrated systems simultaneously promoting information quality, dissemination efficiency, and public health value.Further research that directly evaluates platform recommendation mechanisms and their impact on health information dissemination is warranted to validate the proposed explanatory frameworks.

## Acknowledgments

We would like to thank the staff of the Neurosurgery Research Team at Shaoyang College Affiliated First Hospital for their help and support.

## Author contributions

**Conceptualization:** Can Luo.

**Data curation:** Can Luo.

**Formal analysis:** Xiong Deng.

**Funding acquisition:** Xiong Deng.

**Resources:** Song Tian.

**Software:** JieYao Xia, Song Tian.

**Supervision:** JieYao Xia, Song Tian.

**Validation:** JieYao Xia.




