# Supplementary material for: A multi-platform comparison and machine learning associative analysis study on the information dissemination effect of short videos for cerebral hemorrhage: A cross-sectional study based on TikTok and Bilibili
Source: Medicine (Baltimore). 2026 Aug 21;105(34):e50408. doi: 10.1097/MD.0000000000050408 (PMC13502860; doi:10.1097/MD.0000000000050408)
Supplement: Supplementary file 1 [file medi-105-e50408-s001.docx]

Table S1. Inter-rater Reliability Results

| **Continuous Variables** | | | |
| --- | --- | --- | --- |
| **Variable** | **ICC** | **95% CI** | **p-value** |
| **mDISCERN** | 0.838 | [0.791, 0.875] | < 0.001* |
| **PEMAT-Actionability** | 0.909 | [0.882, 0.931] | < 0.001* |
| **PEMAT-Understandability** | 0.911 | [0.884, 0.932] | < 0.001* |
| **GQS** | 0.897 | [0.740, 0.842] | < 0.001* |
| **Categorical Variables** | | | |
| **Variable** | **Kappa** | | **p-value** |
| **Author Type** | 0.925 | | < 0.001* |
| **Content Type** | 0.878 | | < 0.001* |
| **Disease Dimension** | 0.943 | | < 0.001* |

*P＜0.05
