# Supplementary material for: A multi-platform comparison and machine learning associative analysis study on the information dissemination effect of short videos for cerebral hemorrhage: A cross-sectional study based on TikTok and Bilibili
Source: Medicine (Baltimore). 2026 Aug 21;105(34):e50408. doi: 10.1097/MD.0000000000050408 (PMC13502860; doi:10.1097/MD.0000000000050408)
Supplement: Supplementary file 2 [file medi-105-e50408-s002.docx]

Table S2. Machine Learning Model Performance Metrics

| **Model** | **AUC (95% CI)** | **F1 (95% CI)** | **ACC (95% CI)** | **PPV (95% CI)** | **Recall (95% CI)** |
| --- | --- | --- | --- | --- | --- |
| **Random Forest** | 0.802 (0.776-0.878) | 0.405 (0.291-0.520) | 0.729 (0.689-0.768) | 0.469 (0.408-0.530) | 0.387 (0.165-0.609) |
| **AdaBoost** | 0.811 (0.755-0.889) | 0.458 (0.279-0.636) | 0.739 (0.675-0.803) | 0.488 (0.362-0.614) | 0.467 (0.186-0.747) |
| **LightGBM** | 0.834 (0.752-0.876) | 0.486 (0.393-0.578) | 0.742 (0.684-0.801) | 0.513 (0.400-0.626) | 0.493 (0.270-0.717) |
| **GBDT** | 0.794 (0.750-0.862) | 0.436 (0.291-0.580) | 0.732 (0.672-0.792) | 0.480 (0.337-0.623) | 0.427 (0.188-0.665) |
| **XGBoost** | 0.833 (0.742-0.860) | 0.467 (0.360-0.575) | 0.746 (0.668-0.823) | 0.526 (0.376-0.676) | 0.440 (0.301-0.579) |
| **SVM** | 0.788 (0.689-0.899) | 0.421 (0.153-0.690) | 0.708 (0.643-0.774) | 0.396 (0.255-0.538) | 0.413 (-0.034-0.861) |
| **Logistic Regression** | 0.828 (0.715-0.857) | 0.479 (0.324-0.633) | 0.756 (0.679-0.833) | 0.539 (0.394-0.683) | 0.453 (0.256-0.651) |
| **Decision Tree** | 0.648 (0.694-0.850) | 0.491 (0.358-0.625) | 0.756 (0.660-0.852) | 0.547 (0.369-0.725) | 0.453 (0.345-0.561) |
| **Gaussian NB** | 0.827 (0.667-0.849) | 0.577 (0.520-0.634) | 0.715 (0.670-0.761) | 0.468 (0.422-0.513) | 0.773 (0.574-0.973) |
